# Observing the restriction of another person: vicarious reactance and the role of self-construal and culture

**DOI:** 10.3389/fpsyg.2015.01052

**Published:** 2015-08-04

**Authors:** Sandra Sittenthaler, Eva Traut-Mattausch, Eva Jonas

**Affiliations:** ^1^Division of Economic and Organizational Psychology, Department of Psychology, University of Salzburg, Salzburg, Austria; ^2^Division of Social Psychology, Department of Psychology, University of Salzburg, Salzburg, Austria

**Keywords:** (vicarious) reactance, restrictions, self-construal, culture

## Abstract

Psychological reactance occurs in response to threats posed to perceived behavioral freedoms. Research has shown that people can also experience vicarious reactance. They feel restricted in their own freedom even though they are not personally involved in the restriction but only witness the situation. The phenomenon of vicarious reactance is especially interesting when considered in a cross-cultural context because the cultural specific self-construal plays a crucial role in understanding people’s response to self- and vicariously experienced restrictions. Previous studies and our pilot study (*N* = 197) could show that people with a collectivistic cultural background show higher vicarious reactance compared to people with an individualistic cultural background. But does it matter whether people experience the vicarious restriction for an in-group or an out-group member? Differentiating vicarious-in-group and vicarious-out-group restrictions, Study 1 (*N* = 159) suggests that people with a more interdependent self-construal show stronger vicarious reactance only with regard to in-group restrictions but not with regard to out-group restrictions. In contrast, participants with a more independent self-construal experience stronger reactance when being self-restricted compared to vicariously-restricted. Study 2 (*N* = 180) replicates this pattern conceptually with regard to individualistic and collectivistic cultural background groups. Additionally, participants’ behavioral intentions show the same pattern of results. Moreover a mediation analysis demonstrates that cultural differences in behavioral intentions could be explained through people’s self-construal differences. Thus, the present studies provide new insights and show consistent evidence for vicarious reactance depending on participants’ culturally determined self-construal.

## Introduction and Theory

“*Untold are the hardships of man. Yet nothing worse may befall him but the loss of freedom*.”

Ho Chi Minh

Freedom is one of our most important values, even on a daily basis. We want to be free in thought and behavior, and we wish to be without restrictions. Are these thoughts about freedom universal across cultures or are there cultural differences? In order to answer this question, it seems reasonable to consider previously established research on culture dependent patterns of thinking and perception, e.g., different cognitive styles (Markus and Kitayama, 1991; Nisbett et al., 2001; Varnum et al., 2008, 2010). Thus, studies have shown, for example that Croatians and East Asians think in a more holistic fashion than U.S. Americans and other Westerners. This means that their cognition among other things is characterized by thematic and family-resemblance-based categorization of objects (Nisbett et al., 2001; Varnum et al., 2008). The origin of these cultural differences is traceable to marked differences in interpersonal social orientation. Following Varnum et al. (2010), people differ with regard to possessing a more independent versus interdependent social orientation. Interdependently-orientated people primarily define their self-construal in relation to others: they strive to be part of a group, seek group harmony, and consequently seem to feel free in belonging to a group and following group beliefs. The interdependent view is dominant in people from collectivistic cultures, such as those from Asia and Africa (Markus and Kitayama, 1991; Savani et al., 2008). By contrast, independently-orientated people are characterized by individuality and independence from others. The independent view is most clearly exemplified in individualistic cultures, such as in North America, as well as in many Western European cultures. Statements such as “being free from others” are typical for individualistic cultures (Markus and Kitayama, 1991; Triandis, 1995). As cultures differ with regard to their perception of freedom, they might also differ with regard to their thinking and behaving when this freedom is threatened. Indeed, previous research in the context of reactance theory (Brehm, 1966) has shown that it is important for people from an individualistic cultural background (e.g., European Americans) not to be restricted in their personal freedom (e.g., regarding their personal wishes), whereas for people with a collectivistic cultural background (e.g., Asian Americans), it is more important that their group’s freedom is not restricted (Jonas et al., 2009). Moreover, research not only reveals that there are cultural differences with regard to the experience of reactance, but also that people from a collectivistic cultural background (i.e., Eastern Europeans) show more “vicarious reactance,” i.e., they react more strongly when observing the restriction of another person (Sittenthaler and Jonas, 2012).

In the current paper, the main focus was to investigate the specific role of self-construal and culture when witnessing the restrictions to freedom of another person who belongs to one’s in-group versus to an out-group. What is happening in people with an independent vs. interdependent self-construal, when observing the restriction of another person’s freedom and does it make a difference whether this person is an in-group versus an out-group member? Do people with a more interdependent self-construal experience more vicarious reactance for in-group vs. out-group members than people with a more independent self-construal? We expect to find considerable cultural differences due to differences in peoples’ self-construal.

### Freedom and (Vicarious) Reactance Theory

Reactance theory emphasizes the importance of “specific” individual freedoms and defines conditions under which people react against threats to these freedoms. Reactance is defined as a motivational state with the goal to reestablish the threatened freedom. As reactance is a motivational state it is more than just an emotion such as the feeling of frustration. As a motivational process it should energize and direct behavior toward a positive stimuli or away from a negative stimuli (Roseman, 2009). Nevertheless, reactance may also have applications in explaining concepts such as frustration, social power, and compliance (Brehm, 1966; Donnell et al., 2001). In order to get rid of the unfavorable motivational state of reactance, people can strive to reestablish the threatened freedom directly, e.g., by continuing a behavior, that has been prohibited, or indirectly, e.g., by boycotting another request by the authority that has eliminated one’s autonomy of freedom (Brehm, 1966, 1972; Worchel and Brehm, 1971; Wicklund, 1974; Brehm and Brehm, 1981). A direct method of reestablishing one’s freedom is to engage in the proscribed behavior. For instance, interest in viewing violence in television is increased by reading labels that warn of violent content (Bushman and Stack, 1996), and choice of unhealthy food products like sweet granola bars is increased after reading recommendations for healthier alternative brands (Fitzsimons and Lehmann, 2004). Reactance can also be aroused by product unavailability which motivates people to get the unavailable product (Clee and Wicklund, 1980). Similarly, social influence attempts can backfire (i.e., boomerang effect), in that pressure toward the requested change may induce the person to move in the direction opposite from the influence effort (e.g., Silvia, 2005, 2006).

Beside the broad reactance research, Miron and Brehm (2006) first raised the question of whether reactance could also be experienced on behalf of another person whose freedom of choice is threatened but they did not provide empirical evidence for their idea of “empathic reactance.” However, research concerning different emotions suggests that people can experience vicarious emotions, such as empathic distress or vicarious sympathy (Batson et al., 1987; Ortony et al., 1988; Eisenberg et al., 1991; Scherer, 1998). Furthermore, people can feel vicariously ashamed, guilty or retributive when observing another’s wrongdoing or aggressive behavior in a specific situation (Lickel et al., 2005, 2006). However, related to vicarious “reactant feelings,” Worchel et al. (1974) and Andreoli et al. (1974) described a similar kind of experiencing reactance on behalf of another person in the sense of a perspective-taking point of view. They tried to find out how the observing person thinks about the feelings of the restricted person—however, they did not investigate whether the observer’s own feelings change observing the restriction of another person. Research from our lab (Sittenthaler et al., 2015, in preparation) has shown that people can indeed experience vicarious reactance, when perceiving someone else’s freedom as threatened. However, different processes seem to be activated during self-experienced vs. vicariously experienced reactance. When we experience reactance ourselves, physiological reactions as measured by pulse rate have been found to be stronger for self-experienced as compared to vicarious reactance. In contrast, vicarious reactance is affected by “cognitive load” tasks (e.g., remembering seven digits), suggesting that cognitive processes are important whereas emotional arousal plays less of a role compared to self-experienced reactance, which is primarily affected by “emotional feelings-inducing” tasks (e.g., thinking of a great summer day). Taking together this body of research we suggest a combined dual-process and intertwined-process model explaining the emotional and cognitive processes of self- and vicarious-reactance (Sittenthaler et al., 2015, in preparation).

### Vicarious Reactance and the Role of Culture

“Vicarious reactance” seems to be an especially interesting topic to be examined in a cultural context. A cross-cultural look might help to better understand the phenomenon of vicarious reactance in finding differences concerning vicarious reactance values depending on different cultural background groups. Although reactance is typically considered to be pan-cultural, there seem to be systematic variations based on the cultural background. In a series of studies, Jonas et al. (2009) could show that there are differences in the self-experience of reactance between persons with Eastern versus Western cultural backgrounds. They found that although collectivists reacted to a threat addressing their individual freedom (e.g., a personal good) with less reactance (compared to individualists), they showed strong reactance when the threat addressed a collective freedom (e.g., a common good). Furthermore previous research revealed that people with a collectivistic cultural background experienced more vicarious reactance compared to people with an individualistic cultural background. Steindl and Jonas (2012) and Sittenthaler and Jonas (2012) performed studies on vicarious reactance among collectivists from the Philippines and other Eastern European countries (e.g., the Czech Republic, Romania, Russia) and found that the cultural background plays an important role in understanding different reactance effects. To replicate those interesting findings we conducted a pilot study using a different cultural subgroup.

### Pilot Study

We recruited Arabic immigrants living in Austria as participants for our study. Former research in individualistic countries showed that Muslim participants immigrated in individualistic countries such as the United States (Barry et al., 2000) or France (Croucher et al., 2008) indeed have a more interdependent self-construal. In a similar way, Barry (2005) found that Arabic immigrants (approximately 84%, described themselves as Muslim) were more likely to have an interdependent than an independent self-construal. Muslims emphasize the primary role of family in their life, relationships as well as stress-connectedness and social context. Thus they belong to the group of collectivists (Jameela, 1967; Hasan, 2001). On the other hand, people from Germany and Austria have a similar high I-C Index ( = individualism-collectivism Index) compared to the United States where higher numbers indicate more individualism (Hofstede, 1997; Suh et al., 1998; Schimmack et al., 2002). Thus German and Austrian people could be assigned to the group of individualists. Measuring the self-construal (Triandis and Gelfand, 1998) in our study we could confirm those findings, our Arabic participants (*M* = –1.16, SD = 0.65) were more collectivistic compared to German and Austrian participants (*M* = –0.44, SD = 0.68), *t*(195) = –7.35, *p* < 0.001. A negative value indicated relatively more interdependent self-construal and a positive value indicated relatively more independent self-construal. For that reason, we expected that participants with a collectivistic cultural background would experience more vicarious reactance than participants with an individualistic cultural background. We also assumed that participants with an individualistic cultural background would show more reactance being self-restricted than being vicariously-restricted. The pilot study consisted of a 3 (restriction: self vs. vicarious vs. control condition) × 2 (cultural background: collectivistic vs. individualistic) factorial between subjects design. Participants were randomly assigned to the three experimental restriction conditions. Participants were 197 students and employees living in Salzburg, Austria. 123 participants had an individualistic cultural background [30 male and 92 female, 1 missing value, mean age 25.17 years (SD = 7.78)] and 74 participants had a collectivistic cultural background [15 male and 59 female, mean age 26.12 years (SD = 8.47)]. We used a scenario, manipulating reactance. In the self-restricted condition, participants were asked to imagine that they were searching a new job in a famous company and the personnel manager of the company was not willing to invite them to a job interview for no apparent reason. In the vicariously-restricted condition, the volunteers were asked to think of a friend experiencing the same situation. In the control condition, the students were asked to imagine that s/he would get the job without experiencing any restrictions. After participants had read the scenario, we assessed participants’ feeling of reactance (“experience of reactance”: α = 0.93, 6 items, e.g., “To what extent do you perceive the reaction of the personnel manager as a restriction of freedom?” and “How much pressure do you feel as a result of his reaction?”; see Jonas et al., 2009, as well as “behavioral intentions”: α = 0.84, 7 items, e.g., “How strong is your wish to complain about the personnel manager?” and “Would you like to fight against the personnel managers behavior?”). Answers were given on a 5-point Likert-type scale from 1 (*not at all*) to 5 (*very much*)^1^.

We ran a 3 (restriction: self vs. vicarious vs. control condition) × 2 (cultural background: collectivistic vs. individualistic) analysis of variance (ANOVA) on the experience of reactance measure and found the predicted cultural background × restriction interaction, *F*(2, 191) = 3.11, *p* = 0.047, ${\text{η}}_{p}^{2}$ = 0.03.^2^ The follow-up simple effects analyses within the vicariously-restricted condition supported our hypothesis, that participants with a collectivistic cultural background experienced more vicarious reactance compared to participants with an individualistic cultural background, *F*(1, 191) = 12.83, *p* < 0.001, ${\text{η}}_{p}^{2}$ = 0.06. With regard to the self-restricted, *F*(1, 191) < 1, *p* = 0.974, ${\text{η}}_{p}^{2}$ = 0.01, and the control condition *F*(1, 191) < 1, *p* = 0.355, ${\text{η}}_{p}^{2}$ < 0.01, the experience of reactance did not depend on culture. Participants with a collectivistic cultural background, participants being self-restricted experienced about the same level of reactance compared to participants in the vicariously-restricted condition, *p* = 0.105. Participants in the self-restricted and in the vicariously-restricted condition displayed higher reactance values compared to participants in the control condition, *p*s < 0.001. Participants with an individualistic cultural background tended to show more reactance in the self-restricted condition compared to participants in the vicariously-restricted condition, *p* = 0.065. Participants in the self-restricted and in the vicariously-restricted condition displayed higher reactance compared with participants in the control condition, *p*s < 0.01. Means and standard deviations are displayed in Table 1.

**TABLE 1:** **Means and standard deviations for experience of reactance**.

	**“Individualistic cultural background”^a^, ^b^**		**“Collectivistic cultural background”^a^, ^b^**	
	***M***	**SD**	***M***	**SD**
Self-restricted condition condition	4.31 (*n* = 43)	0.67	4.32 (*n* = 25)	0.56
Vicariously-restricted condition	4.01 (*n* = 40)	0.90	4.65 (*n* = 32)	0.55
Control condition	1.48 (*n* = 40)	0.79	1.68 (*n* = 17)	1.02

^a^Individualistic cultural background = Austrian/German participants; Collectivistic cultural background = Arabic participants. ^b^Judgments were made on a 5-point scale with high values indicating high experience of reactance.

Following these results, we could replicate our previous findings showing that people with a collectivistic cultural background showed more vicarious reactance than people with an individualistic cultural background (Sittenthaler and Jonas, 2012). The increased experience of vicarious reactance can now be generalized to diverse collectivistic countries such as the Philippines, Czech Republic, Russia, Romania as well as Arabic countries compared to Germany and Austria. It seems that vicarious reactance is important for people with a collectivistic cultural background because they define their identity mainly via the relatedness with other people or groups of persons. For that reason, they try to maintain the well-being of the group (e.g., Hannover and Kühnen, 2002). Because other people from their group form a part of their self-construal (Markus and Kitayama, 1991) people with a collectivistic cultural background should be more sensitive to vicarious restrictions compared to people with an individualistic cultural background. However, can we really explain these cultural differences with regard to vicarious reactance with differences in people’s self-construal?

### Self-construal Differences

Restrictions of freedom threaten the self-construal of a person as they imply that the person may not be a self- but an other-directed being. By showing reactance, people demonstrate that they do indeed have freedom and thus increase the value of their threatened self by wanting to restore their freedom (Brehm, 1966; Brehm and Brehm, 1981). Former research as described above was able to show crucial cultural differences in what people consider to be important for their subjective feeling of freedom (Jonas et al., 2009).

Cross-cultural studies, however, show that there are important differences in the self-construal of members of collectivistic and individualistic cultural groups (Singelis, 1994; Hong and Chiu, 2001; Kanagawa et al., 2001; Wang, 2001; Kolstad and Horpestad, 2009). These studies show that the proportion of these aspects in the self-construal of a person stands in relation with the more geographical-societal dimension of individualism and collectivism that was first employed by Hofstede (1980). The cultural self-construal distinguishes between independent and interdependent aspects in the human self. People with a collectivistic cultural background tend to have a more interdependent self-construal focus compared to people with an individualistic cultural background. Interdependence and independence are variables that tend to explain differences at an individual level, related to self-perception (e.g., Markus and Kitayama, 1991; Triandis, 1996; Triandis and Gelfand, 1998). More precisely, as stated by Cross et al. (2011), Individualism-Collectivism is a dimension used to describe cultures, whereas the independent-interdependent self-construal describes individuals. Most cultures seem to present a combination of individualism and collectivism qualities and most people hold both independent and interdependent self-construal in different combination and of different content and quality (Holland et al., 2004). The cultural context typically promotes the development of one or the other self-construal more strongly. Thus, more independent self-construals are dominant in Western cultures (e.g., North American, Western European), whereas more interdependent self-construals are dominant in Eastern and Southern cultures (e.g., Asia, Latin America). People with a more independent self-construal focus on personal attributes (e.g., personal desires), which are independent of others. Their self-construal therefore emphasizes the importance of having the freedom to make one’s own choices and expressing one’s own desires and preferences. Individualism emphasizes individual uniqueness, personal autonomy, and independence. Traditionally, persons with a more interdependent self-construal define their identity mainly in the relatedness with other people or groups of people. For that reason, they also highly value relationships with others, as well as the harmony, interdependent cohesion, and well-being of the group (e.g., Hannover and Kühnen, 2002). Americans, for example, endorse the belief related to individual autonomy, whereas Asians endorse the belief related to collective autonomy (Menon et al., 1999). People with a more interdependent self-construal have a stronger focus on other people. The relationship between the self and other people is assumed to be much closer. Siblings, the mother or father, friends, or even co-workers are integrated in their own self (Markus and Kitayama, 1991). Consequently, concerning restrictions of freedom it should be more important to individuals with a more interdependent self that people who are integrated in their self (like siblings, mother, farther, co-workers) are not restricted in their freedoms—this is as if the person’s own freedom were restricted. For this reason, we suggest that the more interdependent a person’s self-construal, the stronger the experience of vicarious reactance should be. Thus, they should show higher reactance when an in-group member—e.g., a good friend—is restricted in his or her freedom. However, people observing the restrictions of people not belonging to their in-group—i.e., an out-group member—should not show high reactance values. Moreover, with regard to self-experienced reactance, we suggest that the more independent a person’s self-construal the stronger should be the experience of reactance compared to vicarious reactance.

### The Present Research

Recent research showed that people can experience self- and vicarious reactance (Sittenthaler and Jonas, 2012; Steindl and Jonas, 2012; Sittenthaler et al., 2015, in preparation). Furthermore, distinguishing between independent and interdependent self-construal seems to be important for explaining people’s self-reactance behavior as differences can be traced back to differences in the self-construal of people (Jonas et al., 2009).

In the present article, we want to further explore cultural background and self-construal differences in experiencing (vicarious) reactance. As described above, people with a more interdependent self-construal indeed have a greater focus on other people. Self-construal differences cannot only be observed between different cultures but also within one and the same culture (Markus and Kitayama, 1991). In consequence, we suggest that people with different self-construal differ in experiencing (vicarious) restrictions. We furthermore suggest that people differ in experiencing vicarious reactance for the in- vs. the out-group. In a series of studies, Graupmann et al. (2012) showed that collectivists experienced reactance in response to a restriction of freedom only when it came from an out-group, but conformity with the restriction when it came from an in-group. These results suggest that it is crucial to specify the social source of a threat to freedom when determining conditions for psychological reactance. In the context of vicarious reactance it might also be crucial to determine whether the observed restricted person is a member of the in- or the out-group. In our previous research we have not differentiated whether the vicariously restricted person is an in- or out-group member of the participant’s group. In the pilot study the vicariously-restricted person was an in-group-member (e.g., a friend). We hypothesize that for people with a more collectivistic cultural background or a more interdependent self-construal, it should be much more important that an in-group member (e.g., friend, sibling, person of the same nationality, etc.) is not affected by restrictions compared to an out-group member (e.g., person from another nationality). We do not expect differences in experiencing in- or out-group vicarious restrictions concerned to people with an individualistic cultural background as well as people with a more independent self-construal.

To test our hypothesis, we conducted two main studies to investigate the phenomenon of vicarious as opposed to self-experienced reactance in different cultural contexts (cultural background and self-construal). Study 1 was conducted to show that there are interesting differences in experiencing vicarious reactance for the in-group or the out-group for the self-construal focus. Moreover, in Study 2, we tried to replicate the results of Study 1 focussing on cultural background differences and, in addition, we assumed to find differences in participants’ behavioral intentions following self- and vicarious restrictions. Moreover we hypothesize that our cultural diverse effects could be explained through peoples’ individual level of self-construal^3^.

## Study 1: Differences in Experiencing Vicarious Reactance for the In- or Out-group and the Role of Self-construal

We expected that people with a more interdependent self-construal show more vicarious reactance for the in-group compared to people with a more independent self-construal. Furthermore, people with an independent self-construal should show higher reactance in experiencing self-restrictions compared to both forms of vicarious (in-group and out-group) restrictions. More precisely, we expected a restriction × self-construal interaction comparing the self-restricted and vicariously-in-group restricted (but not for the vicariously-out-group) condition.

### Materials and Methods

#### Participants and Design

Participants were volunteers (43 males, 115 females, 1 missing value; *N* = 159) recruited at the University of Salzburg, Austria, and in Munich. Participants had a mean age of 31.01 years (SD = 12.74). The experiment was based on three experimental conditions (restriction: self vs. vicarious in-group vs. vicarious out-group). In addition we assessed one individual difference variable (self-construal: independent vs. interdependent) as the independent variable. The participants were randomly assigned to the three restriction conditions.

#### Experimental Procedures

Participants were asked to participate in a 10-min paper-and-pencil study and to complete all questions honestly and silently. The questionnaire started with some general questions about sex, age, and, field of study. After that, participants were asked to reflect upon a “holiday-scenario,” in which their freedom was restricted because they were held up by the police after an accident while on vacation. In the self-restricted condition, they were asked to imagine that they were held up by the police after an accident without any good reasons. In the vicarious in-group condition, they had to imagine a person speaking the same language being held up by the police whereas in the vicarious out-group condition the participants had to imagine the same scenario for a person speaking a foreign language. Consistent with the pilot study, the internal consistency of the reactance scale “experience of reactance” (α = 0.84, 7 items, e.g., “To what extent do you perceive the reaction of the policeman as a restriction of freedom?” and “How much pressure do you feel as a result of his reaction?”) was acceptable, but not for our “behavioral intentions” scale (α = 0.58, 6 items, e.g., “Would you complain about the policeman’s behavior at the police station?”). For this reason we did not further consider this measure for our result section. Responses were made on a scale from 1 = *strongly disagree* to 10 = *strongly agree* in this study. To measure independent and interdependent attitudes and values, participants were then presented with the self-construal-short-scale (Triandis and Gelfand, 1998), which consists of 32 items with a focus either on participant’s independent (8 items such as “My personal identity, independent of others, is very important to me.,” α = 0.78) or interdependent (8 items such as “I feel good when I cooperate with others.,” α = 0.72) self-construal. All responses were made on a scale from 1 = *strongly disagree* to 5 = *strongly agree*. At the end of the study, the volunteers were debriefed and thanked for their participation.

### Results

#### Experience of Reactance

For our measure of “Experience of Reactance,” we ran a univariate analysis of variance (ANOVA) and found a significant main effect of the restriction condition, *F*(2, 156) = 3.49, *p* = 0.033, ${\text{η}}_{p}^{2}$ = 0.04. Participants in the self-restricted condition (*M* = 6.31, SD = 2.01) showed higher experience of reactance scores compared to participants in the vicariously-in-group restricted condition (*M* = 5.46, SD = 2.15) and the vicariously-out-group condition (*M* = 5.31, SD = 2.20). Subsequent *post hoc* analysis showed significant differences between both the vicariously-out-group restricted (*p* = 0.016) and the vicariously-in-group condition (*p* = 0.040) compared with the self-restricted condition. In addition, there was no significant difference between vicariously-out-group restricted and vicariously-in-group restricted condition, *p* = 0.716.

#### Self-construal as Moderator

For further analyses concerning our assumption that people with a more interdependent self-construal show more vicarious reactance than people with a more independent self-construal while people with a more independent self-construal show more self-reactance than vicarious reactance, we followed previous research and created a difference score between the scores on the independent and interdependent sub-scales of the Triandis scale (e.g., Holland et al., 2004; Pöhlmann et al., 2007; Jonas et al., 2009). The correlation between the independent and the interdependent measure was significant, i.e., dependent and not orthogonal constructs [*r*(159) = –0.734, *p* < 0.001]. Consequently, a negative value indicated relatively more interdependent self-construal and a positive value indicated relatively more independent self-construal.

Furthermore, two dummy codes (Dummy1/Dummy2) were created for the three restriction conditions (self-restricted vs. vicariously-in-group restricted vs. vicariously-out-group-restricted). In the following analyses, the self-restricted condition is the reference group (Dummy 1 and Dummy 2 = 0). To test our hypotheses, we conducted a regression analysis. Following Aiken and West (1991) and Cohen et al. (2003), the interaction terms were computed by a multiplication of the dummy variables (self-restricted as reference group) with the z-standardized difference score.

**FIGURE 1:**
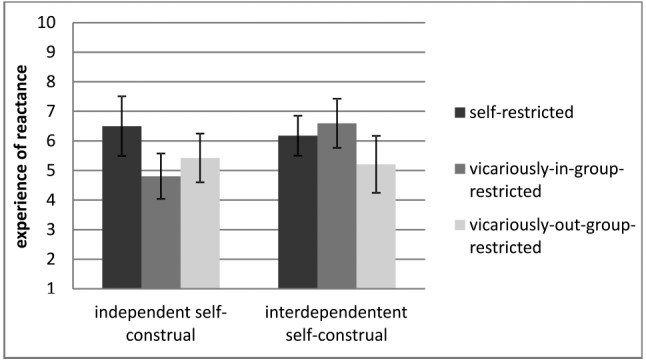
**Study 1: Interactions Restriction × Self-Construal experiencing reactance**.

We found the assumed restriction × self-construal interaction for vicariously-in-group restricted participants compared to the self-restriction condition, β = –0.29, |*t*(157)| = 2.90, *p* = 0.004. As expected, we did not find the restriction × self-construal interaction comparing vicariously-out-group restricted condition to the self-restricted condition, β = –0.08, |*t*(158)| = 0.75, *p* = 0.453. The interactions are illustrated in Figure 1. According to our hypothesis, we assumed that participants holding a more interdependent self-construal should show more experience of reactance when they are restricted vicariously for the in-group, as opposed to participants holding a more independent self-construal. The result pattern displayed in Figure 1 seems to be consistent with this hypothesis. Simple slope analyses were conducted to further analyze the interaction between restriction (self vs. vicariously-in-group-restricted) and self-construal (independent vs. interdependent; Aiken and West, 1991). Simple slope analyses indicated that in the vicariously-in-group-restricted condition, participants with a more interdependent self-construal showed more reactance compared to participants with a more independent self-construal, β = –0.46, |*t*(106)| = 2.96, *p* = 0.004. There was a second significant effect in the self-restriction condition: people with an independent self-construal showed higher reactance scores compared to people with a more interdependent self-construal, β = 0.72, |*t*(106)| = 2.55 *p* = 0.012. Furthermore, participants holding a more independent self-construal (+1 SD) showed more reactance when they were self-restricted compared to vicariously-in-group-restricted, β = –0.45, |*t*(106)| = 3.50, *p* = 0.001, whereas participants holding a more interdependent self-construal (–1 SD) did not differ in their experience of reactance being self-restricted or vicariously-in-group-restricted, β = 0.15, |*t*(106)| = 1.04, *p* = 0.300.

Comparing vicariously-in-group-restricted participants and vicariously-out-group (vicariously-in-group-restricted participants as reference group with two more dummies 3 and 4) a second significant interaction was found, β = 0.26, |*t*(158)| = 2.17, *p* = 0.032. Simple slope analyses were conducted to further analyze the interaction between restriction (vicariously-in-group-restricted vs. vicariously-out-group-restricted) and self-construal (independent vs. interdependent). Simple slope analyses indicated that in the vicariously-in-group-restricted condition participants with a more interdependent self-construal showed more reactance compared to participants with a more independent self-construal, β = –0.86, |*t*(103)| = –2.57, *p* = 0.012. There was no significant effect in the vicarious-out-group-restriction condition: people with an independent self-construal showed nearly the same reactance scores compared to people with a more interdependent self-construal, β = –0.00, |*t*(103)| = –0.03, *p* = 0.974. Furthermore, participants holding a more interdependent self-construal (– 1 SD) showed more reactance when they were vicariously-in-group-restricted compared to vicariously-out-group-restricted, β = –0.32, |*t*(103)| = –2.11, *p* = 0.037, whereas participants holding a more independent self-construal (+1 SD) did not differ in their experience of reactance being vicariously-out-group or vicariously-in-group-restricted, β = 0.13, |*t*(103)| = 0.93, *p* = 0.353.

### Discussion

Study 1 could show that people holding a more interdependent self-construal experienced more vicarious reactance with regard to the in-group compared to people with a more independent self-construal. This replicates our pilot study findings showing that people with a more interdependent self-construal or a collectivistic cultural background are much more concerned by vicarious restrictions. In addition people with a more interdependent self-construal showed more reactance when the restriction originated in an in-group compared to an out-group member. Furthermore, people with a more independent self-construal showed more reactance experiencing self- restrictions compared to vicarious restrictions. Moreover, people with a more independent self-construal were much more affected by self-restrictions compared to people with a more interdependent self-construal.

Considering these results, it seems that people indeed distinguish as to whether the other person who is vicariously restricted is an in-group or an out-group member. Brewer and Gardner (1996) found that people seek to maintain a positive social identity by giving positive value to their own group (in-group) and distinguishing their group from other groups (out-groups). In a meta-analysis of conformity research across cultures, Bond and Smith (1996) emphasized the influence of group context on the collectivist’s tendency to conform with group members. They pointed out that people with collectivistic cultural background would thus be expected to conform more to members of the in-group, and less to members of an out-group than individualists. This is interesting considering our results that people with a more interdependent self-construal showed higher vicarious reactance values for a restricted in-group-member.

In the following Study 2, we wanted to replicate the pattern of results from Study 1 when comparing people with a collectivistic cultural background to people with an individualistic cultural background. Furthermore, we looked at behavioral intentions in addition to experience of reactance.

## Study 2: Differences in Experiencing Vicarious Reactance for the In- and Out-group and the Role of Culture

In Study 2, we wanted to find out whether people with a more collectivistic or individualistic cultural background also differentiate between an in- vs. an out-group member when experiencing vicarious reactance. Croatians and a few Bosnians as well as Germans/Austrians were asked to imagine a holiday situation being held up by the police after an accident. A cross-cultural study by Tavakoli et al. (2003) showed that Croatian people scored significantly higher in both Power Distance and Uncertainty Avoidance while the United States scored higher in both Individuality and Masculinity. The United States scored extremely high on the Individuality dimension—in fact, they got the highest score of those cultures studied by Hofstede (1997)—while Croatia scored moderately on the individualism dimension, being more of a collectivistic culture. Compared to that, former research also showed that people of the United States can be compared to West German people concerning their individualism score (Suh et al., 1998; Schimmack et al., 2002). Measuring the self-construal (Triandis and Gelfand, 1998) in our study we could confirm those findings, our Croatian participants (*M* = – 0.69 SD = 0.80) were more collectivistic compared to German/Austrian participants (M = – 0.37 SD = 0.78), *t*(178) = 2.662, *p* = 0.008. As described in our pilot study a negative value indicated relatively more interdependent self-construal and a positive value indicated relatively more independent self-construal.

For that reason, we assumed that people with a more collectivistic cultural background experience more vicarious reactance and show more behavioral intentions for the in-group compared to people with a more individualistic background. We also expect to find that the restriction × cultural background interaction on reactance behavior is mediated by experience of reactance.

### Materials and Methods

#### Participants and Design

Study 2 consisted of a 3 (restriction: self vs. vicarious in-group vs. vicarious out-group) × 2 (cultural background: collectivistic vs. individualistic) factorial between subjects design. Participants (*N* = 180) were randomly assigned to the experimental restriction conditions. Austrian and German participants (*n* = 90) were mainly recruited in Salzburg, Austria. Austrian and German participants consisted of 54 male and 36 female with a mean age of 35.39 (SD = 11.01). Croatian and a few Bosnian participants (*n* = 90) were recruited via visiting people in different areas of Croatia and via the internet. Participants consisted of 46 male and 44 female with a mean age of 34.41 (SD = 11.55). Participants recruited for this study were employees (e.g., technicians, workers, painters).

#### Experimental Procedures

Participants were invited to take part in a paper-and-pencil questionnaire. First participants were asked to complete demographic questions regarding their sex, age, and work. After that, participants were asked to reflect upon the “holiday-scenario,” restricting their freedom through being held up by the police after an accident while on vacation in Spain, as already described in Study 1. For the Croatian and Bosnian participants the scenario and the whole questionnaire were translated and also retranslated. Consistent with Study 1 and the pilot study, the internal consistency of the “experience of reactance scale was acceptable (α = 0.85, 7 items).” The internal consistency of our additional measured “behavioral intentions” was also acceptable, as we reformulated the items used in Study 1 as well as generated “better” items for the specific used scenario (α = 0.83, 7 items, e.g., “To what extent would you describe this policeman as incompetent to other people?” How strong is your wish to complain about the policeman at the next police office? and “Would you like to ruin this police’s reputation by publishing a negative review on a respected newspaper?”). Answers were given on a 5-point Likert-type scale from 1 (*not at all*) to 5 (*very much*).

### Results

#### Experience of Reactance

We ran a 3 (restriction: self vs. vicarious in-group vs. vicarious out-group) × 2 (cultural background: collectivistic vs. individualistic) analysis of variance (ANOVA) on the experience of reactance measure and revealed a significant overall main-effect for the cultural background, *F*(1, 174) = 15.64, *p* < 0.001, ${\text{η}}_{p}^{2}$ = 0.08, indicating that participants with a collectivistic cultural background (*M* = 3.58, SD = 0.91) show higher experience of reactance values than participants with an individualistic cultural background (*M* = 3.03, SD = 0.99). We could also find a significant main-effect for the restriction condition, *F*(2, 174) = 3.04, *p* = 0.050, ${\text{η}}_{p}^{2}$ = 0.03, indicating that participants in the self-restricted (*M* = 3.48, SD = 0.99) and the vicariously-in-group-restricted conditions (*M* = 3.37, SD = 1.07) showed higher experience of reactance scores than participants in the vicariously-out-group-restricted condition (*M* = 3.07, SD = 0.86). Subsequent *post hoc* analysis showed (marginal) significant differences between both the self-restricted (*p* = 0.019) and the vicariously-in-group-restricted condition (*p* = 0.080) compared to the vicariously-out-group-restricted condition. In addition, there was no significant difference between the self-restricted and the vicariously-in-group-restricted condition, *p* = 0.539.

However, most importantly, we found the predicted cultural background × restriction interaction, *F*(2, 174) = 3.87, *p* = 0.023, ${\text{η}}_{p}^{2}$ = 0.04. The follow-up simple effects analyses within the vicariously-in-group-restricted condition supported our hypothesis, that participants with a collectivistic cultural background experience more vicarious reactance for their in-group compared to participants with an individualistic cultural background, *F*(1, 174) = 16.88, *p* < 0.001, ${\text{η}}_{p}^{2}$ = 0.09. Moreover, within the vicariously-out-group-restricted condition we revealed a significant effect, *F*(1, 174) = 6.40, *p* = 0.012, ${\text{η}}_{p}^{2}$ = 0.04, showing that people with a collectivistic cultural background show more vicarious reactance for the out-group compared to people with an individualistic cultural background. In the self-restricted condition, the cultural background had no effect on experience of reactance, *F*(1, 174) < 1, *p* = 0.832, ${\text{η}}_{p}^{2}$ < 0.01. Within the Collectivistic cultural background group, participants in the vicariously-in-group-restricted condition displayed higher reactance values compared with participants in the vicariously-out-group-restricted condition, *p* = 0.043. Participants being self-restricted experienced approximately the same level of reactance compared to participants in the vicariously-out-group-restricted condition, *p* = 0.602 and the vicariously-in-group-restricted condition, *p* = 0.132. Following the Individualistic cultural background group, participants in the self-restricted condition showed more reactance than participants in the vicariously-in-group-restricted condition, *p* = 0.018 and in the vicariously-out-group-restricted condition, *p* = 0.005. Participants with an individualistic cultural background showed the same level of reactance in the vicariously-in-group-restricted condition and in the vicariously-out-group-restricted condition, *p* = 0.649. Means and standard deviations are displayed in Table 2.

**TABLE 2:** **Means and standard deviations for experience of reactance**.

	**“Individualistic cultural background”^a^, ^b^**		**“Collectivistic cultural background”^a^, ^b^**	
	***M***	**SD**	***M***	**SD**
Self-restricted condition	3.45 (*n* = 30)	0.93	3.50 (*n* = 30)	1.06
Vicariously-in-group-restricted Condition	2.88 (*n* = 30)	1.03	3.86 (*n* = 30)	0.87
Vicariously-out-group-restricted Condition	2.77 (*n* = 30)	0.90	3.38 (*n* = 30)	0.71

^a^Individualistic cultural background = Austrian/German participants; Collectivistic cultural background = Croatian/Bosnian participants. ^b^Judgments were made on a 5-point scale with high values indicating high experience of reactance.

#### Behavioral Intentions

We ran a second 3 (restriction: self vs. vicarious in-group vs. vicarious out-group) × 2 (cultural background: collectivistic vs. individualistic) analysis of variance (ANOVA) on the behavioral intentions measure and revealed a significant overall main-effect for the cultural background, *F*(1, 174) = 17.53, *p* < 0.001, ${\text{η}}_{p}^{2}$ = 0.09, indicating that participants with a collectivistic cultural background (*M* = 2.92, SD = 0.96) display higher values concerning the behavioral intentions than participants with an individualistic cultural background (*M* = 2.36, SD = 0.88). We were not able to find a significant main-effect for the restriction condition, *F*(2, 174) = 2.24, *p* = 0.109, ${\text{η}}_{p}^{2}$ = 0.03.

However, most importantly, we found the predicted cultural background × restriction interaction, *F*(2, 174) = 3.87, *p* = 0.023, ${\text{η}}_{p}^{2}$ = 0.04. The follow-up simple effects analyses within the vicariously-in-group-restricted condition supported our hypothesis, that participants with a collectivistic cultural background showed more vicarious behavioral intentions for their in-group compared to participants with an individualistic cultural background, *F*(1, 174) = 21.57, *p* < 0.001, ${\text{η}}_{p}^{2}$ = 0.11. Within the vicariously-out-group-restricted condition we revealed a marginal effect, *F*(1, 174) = 2.85, *p* = 0.093, ${\text{η}}_{p}^{2}$ = 0.02, showing that people with a collectivistic cultural background tended to show more vicarious behavioral intentions for the out-group compared to people with an individualistic cultural background. In the self-restricted condition, the culture had no effect on behavioral intentions, *F*(1, 174) < 1, *p* = 0.360, ${\text{η}}_{p}^{2}$ = 0.01. Within the Collectivistic cultural background group, participants being self-restricted showed about the same level of behavioral intentions compared with participants in the vicariously-out-group-restricted condition, *p* = 0.284 and the vicariously-in-group-restricted condition, *p* = 0.154. Participants in the vicariously-in-group-restricted condition displayed higher behavioral intentions values compared with participants in the vicariously-out-group-restricted condition, *p* = 0.013. Following the Individualistic cultural background group, participants in the self-restricted condition showed more behavioral intentions as participants in the vicariously-in-group-restricted condition, *p* = 0.023 and in the vicariously-out-group-restricted condition, *p* = 0.067. Participants with an individualistic cultural background showed the same level of reactance in the vicariously-in-group-restricted condition and vicariously-out-group-restricted condition, *p* = 0.653. Means and standard deviations are displayed in Table 3.

**TABLE 3:** **Means and standard deviations for behavioral intentions**.

	**“Individualistic cultural background”^a^, ^b^**		**“Collectivistic cultural background”^a^, ^b^**	
	***M***	**SD**	***M***	**SD**
Self-restricted condition	2.68 (*n* = 30)	0.89	2.90 (*n* = 30)	1.09
Vicariously-in-group-restricted Condition	2.15 (*n* = 30)	0.79	3.23 (*n* = 30)	0.96
Vicariously-out-group-restricted Condition	2.25 (*n* = 30)	0.89	2.65 (*n* = 30)	0.74

^a^Individualistic cultural background = Austrian/German participants; Collectivistic cultural background = Croatian/Bosnian participants. ^b^Judgments were made on a 5-point scale with high values indicating high behavioral intentions.

***Mediation mechanisms***

We tested whether the restriction × cultural background interaction on reactance behavior was mediated by experience of reactance. Thus an ANCOVA was performed with reactance behavior as the dependent variable and experience of reactance as the covariate. The interaction term (restriction x cultural background) was reduced from *F*(2, 174) = 3.87, *p* = 0.023, ${\text{η}}_{p}^{2}$ = 0.04 without the mediator to *F*(2, 173) = 1.68, *p* = 0.189, ${\text{η}}_{p}^{2}$ = 0.02, where the mediator (experience of reactance) was significant, *F*(1, 173) = 69.04, *p* < 0.001, ${\text{η}}_{p}^{2}$ = 0.29. Hence, these results support the assumption that experience of reactance mediates the relationship between restriction and cultural background on behavioral intentions with a substantial reduction of the interaction effect size from 0.04 to 0.02 (approximately 50%).

Finally, we analyzed whether the effect observed for participants from the two different cultural backgrounds being self vs. vicariously-in-group restricted on behavioral intentions could be explained through peoples’ individual level of self-construal (mediator) using PROCESS (model 4, Hayes, 2013, p. 445). The analyses revealed a significant influence of cultural background on behavioral intentions, *B* = 0.75, SE = 0.18, *t*(110) = 4.20, *p* < 0.001, and the mediator participants’ self-construal, *B* = -0.68, SE = 0.21, *t*(110) = -3.31, *p* = 0.001. Subsequent analysis of the influence of the mediator on behavioral intentions showed a significant regression weight, *B* = 0.28, SE = 0.08, *t*(110) = 3.50, *p* < 0.001, indicating considerable influence of mediator on behavioral intentions. When finally examining the influence of the cultural background and the mediator participants’ self-construal on behavioral intentions concurrently, the effect of cultural background still remained significant, *B* = 0.94, SE = 0.18, *t*(110) = 5.25, *p* < 0.001. The indirect effect of the cultural background on behavioral intentions through the mediator participants’ self-construal was highly significant as indicated by the 95% CI (–0.38, –0.07) using 5,000 bootstrap estimations.

### Discussion

Study 2 showed that people with a collectivistic cultural background (Croatian) experienced significantly more vicarious reactance for the in-group compared to people with an individualistic cultural background (Austrians/Germans). We assumed that people with a collectivistic cultural background being rather positively associated with the in-group (Triandis et al., 1988) would react strongly when the in-group’s freedom was threatened and would therefore show more vicarious reactance when an in-group member (as part of the group) was restricted in his or her freedom. And indeed this was what our findings confirmed. Furthermore, as stated by Triandis et al. (1988), collectivists generally have higher levels of in-group commitment than individualists. This finding is reflected in our results, our Croatian participants seemed to be much more committed to the person speaking their own language reading the holiday scenario. Therefore they were also able to show more vicarious reactance for this in-group person compared to an out-group person speaking a foreign language.

In addition, participants with an individualistic cultural background showed more self-experienced reactance compared to vicarious reactance. People in individualistic cultures define their self-construal more independently than interdependently from others: the freedom to make one’s own choices and to express one’s own desires and preferences are important (Triandis et al., 1988; Hannover and Kühnen, 2002). As a consequence, they feel significantly more reactance when they are self-restricted. People can also vary in the degree to which they identify with their in-groups (Stangor and Thompson, 2002; Vignoles and Moncaster, 2007). However, people with an individualistic background seem to be less sensitive to vicarious restrictions of an in-group-member. The same result pattern could be shown for our second dependent variable “behavioral intentions.” People with a collectivistic cultural background showed more vicarious behavioral intentions for the in-group compared to people with an individualistic cultural background. An individualistic cultural background also led to higher reactance in participants being self-restricted compared to vicariously-restricted.

Furthermore, the mediation results showed that the behavioral intentions differences which could be observed for the two different cultural groups can be explained via self-construal differences.

## General Discussion

### Discussion of the Results

In the current article, we were interested in investigating self-experienced and vicariously-experienced reactance depending on the cultural background of participants as well as differences in self-construal. We sought to determine whether people with a more interdependent self-construal or with a collectivistic cultural background would show more vicarious reactance (for the in-group) compared to people with a more independent self-construal or an individualistic cultural background. In two studies, we found consistent evidence for vicarious reactance depending on participants’ culturally determined self-construal. People with a more interdependent self-construal generally reacted with more vicarious reactance and at the same time also vicariously for the in-group compared to people with a more independent self-construal (Study 1). The pilot study as well as Study 2 could further demonstrate that people with a collectivistic cultural background showed more vicarious reactance in general as well as for the in-group compared to people with an individualistic cultural background, which was in line with our expectations. In contrast, participants with a more independent self-construal or an individualistic cultural background showed stronger reactance being self-restricted compared to being vicariously-restricted. We were able to demonstrate the differences in self-construal as a result of cultural background.

It seems that when observing other people’s restrictions participants with a more interdependent self-construal or a collectivistic cultural background are much better at feeling with the restricted person and thus show higher vicarious reactance values compared to people with a more independent self-construal or an individualistic cultural background- and perspective-taking seems to be crucial in understanding the phenomenon of vicarious processes as Krach et al. (2011) suggested, linking empathy to vicarious embarrassment in his studies. Extending Miller’s (1987) work, he and his colleagues postulate that it is an empathic process and, accordingly, the capability to represent other people’s inner states that enables observers to experience vicarious processes (i.e., vicarious embarrassment). Previous research claimed that perspective taking is valued more highly by persons with a collectivist orientation compared to people with an individualistic orientation (Markus and Kitayama, 1991; Vorauer and Cameron, 2002). This is supported by a cross-cultural study showing that cultural patterns of interdependence focus attention on the other, causing Chinese to be better perspective takers than Americans (Wu and Keysar, 2007).

Our vicarious reactance research might explain open questions of averseness to foreigners (members of an out-group). As we were able to show in our research, people are mainly affected by restrictions concerning an in-group member. Thus, it is not surprising that in daily life, people tend to help people of their own nationality if they are in trouble (e.g., if they are being restricted) rather than to help people of foreign nations. Furthermore aggressions against out-group members have often been observed (e.g., Struch and Schwartz, 1989). In future research, it would be interesting to develop a tool against xenophobic aggressions, e.g., an intervention, blocking people’s negative cogitation building on our findings that vicarious reactance ought to be a cognitive process (Sittenthaler et al., 2015, in preparation). A promising approach is the idea that people should develop “cognitive empathy,” described by Stephan and Finlay (1999): “Cognitive empathy may reduce prejudice because it leads people to see that they are less different from members of the other group than they thought they were. [...] The feelings of threat engendered by concerns over differences in values, beliefs, and norms, misperceptions of realistic conflict, and anxiety over interacting with members of the out-group may all be dissolved by learning to view the world from the perspective of out-group members. [...] Understanding the ways that others view the world has the potential to make them seem less alien and frightening and thus to break down the perceived barriers between the in-group and the out-group” (p.735). As stated by Stephan and Finlay (1999), an emotional affect such as empathy can easily be connected with cognitive processes. In experiencing vicarious reactance people also “feel” with the restricted person but also have to “think” about the situation they observe. Future research should further address our proposed combined dual-process and intertwined-process model explaining vicarious reactance processes (Sittenthaler et al., 2015, in preparation) to get the whole picture of the phenomenon of vicarious reactance.

### Limitations

Even though the present studies provide valuable new insights, they also have important limitations.

One major concern is that we unfortunately did not have equal alternatives in our reactance arousing scenarios. Thus one could question whether reactance or another construct as frustration is the main driving force in our studies. As described in the Footnotes, we controlled for negative affect (using the negative PANAS Scale). Although we found moderate significant correlations with our reactance measures in Study 2 (indicating an overlap between both constructs) we only found the specific culture × restriction interaction effect on our reactance measure but not on the negative PANAS measure (*p* = 0.495). Moreover, in a related study, in which a choice option in the work context was eliminated (equal office rooms) either for the participant herself/ himself or a co-worker, we also found vicarious reactance effects for the collectivistic subsample. In contrast to Western-Europeans East Asians expressed heightened attractiveness for the option when it had been excluded for their coworker (Graupmann and Jonas, 2015, in preparation). This conceptually replicates the findings of the current studies and thus renders an alternative explanation via frustration unlikely. In addition, we also conceptually replicated our results in a series of other studies we did with young children (Austrian and Arabic cultural background) using different sweets and little cuddly toys as alternatives and in which we took away the third ranked alternative. In these studies children indeed showed attractiveness change for the third alternative as well as experience of reactance on a child-modified experience of reactance scale used in this paper. Moreover and most importantly children showed much more vicarious reactance observing the restriction of an in-group compared to an out-group member (Sittenthaler and Jonas, 2015, in preparation). Taken together, these results underline our assumption that reactance (and not frustration per se) plays a crucial role in understanding culture dependent effects on people’s responses to threats to their freedom.

Another limitation results from the fact that in our studies, we only worked with participants with Arabic and Croatian as well as German and Austrian background. However, these results add to previous studies on vicarious reactance among people from the Czech Republic, Romania, and the Philippines. But to have a better understanding of cultural issues, research needs to cover more geographically extended areas. The degree of experiencing self- and vicarious reactance may vary from country to country depending on the culture-specific self-construal. It should also be noted that it would be interesting for future research to examine more participants with a typical individualistic and collectivist background (e.g., Japanese or American people) in order to generalize the findings beyond the cases studied in our article and to test whether this would result in even stronger differences. Regarding cultural differences and self-construal differences, Brewer and Chen (2007) suggested a theoretical framework and conceptual clarification of individualism and collectivism and distinguished relational, collective, and individual selves drawing on a conceptualization of Brewer and Gardner (1996). In order to get a better understanding of cultural differences it is important to get a clearer picture of the different determinants of people’s self-construal. In future research work a main focus should be on finding a better measure method to determine the self-construal instead of using the Triandis self-construal scale. Moreover, one should also be aware of cultural changes over time interpreting culture-sensitive research results, as Podrug et al. (2014) recently showed in a cross-cultural study that the most significant cultural change is the shift toward individualism, mainly in Brazil, Croatia, and Serbia.

We are also aware that we need to interpret our results with caution, because all experiments were based on hypothetical scenarios. However, with the results showing a consistent pattern among different scenarios “job-scenario” and “holiday-scenario,” we are confident that they could also be replicated with real behavior in future research.

### Conclusion

In sum, our research shows that people with individualistic compared to collectivistic cultural backgrounds indeed experience self-restrictions and vicarious restrictions differently. People with a collectivistic cultural background or a more interdependent self-focus are more affected when experiencing vicarious restrictions of other persons, especially in-group members. In contrast, people with an individualistic cultural background or a more independent self-construal are much more affected by self-restrictions and show high experience of reactance as well as behavioral intentions scores compared to vicarious restrictions. An important follow-up question would be which further mediators could explain the occurrence of vicarious reactance depending on different self-construal and culture focus. The investigation of this and other questions will be the subject of further research.

### Conflict of Interest Statement

The authors declare that the research was conducted in the absence of any commercial or financial relationships that could be construed as a potential conflict of interest.

## Footnotes

1 For explorative analyses we measured the subscales “Victim Sensitivity” and “Observer Sensitivity” from the “Justice Sensitivity Inventory” by Schmitt et al. (2005) in all studies. Furthermore we controlled for negative affect using the negative PANAS Scale by Watson et al. (1988). In our pilot study and Study 1 we tried to figure out the identity of our participants using adapted Venn-diagrams by Schubert and Otten (2002). In Study 2 we used items from the Generalized self-efficacy scale by Schwarzer and Jerusalem (1995) and *outrage* measurement by Batson et al. (2007). In our pilot study we utilized *The Therapeutic Reactance Scale* by Dowd et al. (1991) as well as an adapted version of the *Religion Scale* by Zwingmann et al. (1994).

2 Furthermore, we found a significant main-effect of the restriction manipulation, *F*(2, 191) = 235.88, *p* < 0.001, ${\text{η}}_{p}^{2}$ = 0.71, indicating that participants in the self-restricted (*M* = 4.32, SD = 0.63) and the vicariously-restricted conditions (*M* = 4.29, SD = 0.82) showed higher experience of reactance scores than participants in the control condition (*M* = 1.54, SD = 0.86). Subsequent *post hoc* analysis showed significant differences between both the self-restricted and the vicariously-restricted condition compared to the control condition, *p*s < 0.001. In addition, there was no significant difference between the self-restricted and the vicariously-restricted condition, *p* = 0.938. A second main-effect was found for the cultural background, *F*(1, 191) = 6.24, *p* < 0.013, ${\text{η}}_{p}^{2}$ = 0.03, indicating that participants with a collectivistic cultural background (*M* = 3.85, SD = 1.38) showed higher experience of reactance scores compared to participants with an individualistic cultural background (*M* = 3.29, SD = 1.49). A second ANOVA concerning behavioral intentions did not provide a significant restriction × cultural background interaction effect, *F*(2, 191) < 1, *p* = 0.647, ${\text{η}}_{p}^{2}$ = 0.01.

3 Both main studies as well as the pilot study were conducted before the ethical board at the University of Salzburg was established in 2011. Nevertheless, treatments of the participants in the study were in accordance with APA ethical standards.
