# Erratum: Observing the restriction of another person: vicarious reactance and the role of self-construal and culture

**DOI:** 10.3389/fpsyg.2015.01584

**Published:** 2015-10-09

**Authors:** Frontiers Production Office

**Affiliations:** Frontiers Production Office, FrontiersLausanne, Switzerland

**Keywords:** (vicarious) reactance, restrictions, self-construal, culture

Reason for Erratum:

Due to a typesetting error, in the Results section, values were reported incorrectly in the second paragraph of subsection Experience of Reactance.

*F*_(2, 174)_ = 3.87, should read as: *F*_(2, 174)_ = 3.84.

The publisher apologizes for this mistake. This error does not change the scientific conclusions of the article in any way.

The original article was updated.

